# Flexural Properties and Elastic Modulus of Different Esthetic Restorative Materials: Evaluation after Exposure to Acidic Drink

**DOI:** 10.1155/2019/5109481

**Published:** 2019-02-04

**Authors:** Andrea Scribante, Marco Bollardi, Marco Chiesa, Claudio Poggio, Marco Colombo

**Affiliations:** ^1^Unit of Orthodontics and Paediatric Dentistry, Section of Dentistry, Department of Clinical, Surgical, Diagnostic and Paediatric Sciences, University of Pavia, Pavia, Italy; ^2^Unit of Restorative Dentistry, Section of Dentistry, Department of Clinical, Surgical, Diagnostic and Paediatric Sciences, University of Pavia, Pavia, Italy

## Abstract

**Background:**

Acidic beverages, such as soft drinks, can produce erosion of resin composites. The purpose of the present study was to investigate mechanical properties of different esthetic restorative materials after exposure to acidic drink.

**Methods:**

Nine different composites were tested: nanofilled (Filtek Supreme XTE, 3M ESPE), microfilled hybrid (G-ænial, GC Corporation), nanohybrid Ormocer (Admira Fusion, Voco), microfilled (Gradia Direct, GC Corporation), microfilled hybrid (Essentia, GC Corporation), nanoceramic (Ceram.X Universal, Dentsply De Trey), supranano spherical hybrid (Estelite Asteria, Tokuyama Dental Corporation), flowable microfilled hybrid (Gradia Direct Flo, GC Corporation), and bulk fill flowable (SureFil SDR flow, Dentsply De Trey). Thirty specimens of each esthetic restorative material were divided into 3 subgroups (n=10): specimens of subgroup 1 were used as control, specimens of subgroup 2 were immersed in 50 ml of Coca Cola for 1 week, and specimens of subgroup 3 were immersed in 50 ml of Coca Cola for 1 month. Flexural strength and elastic modulus were measured for each material with an Instron Universal Testing Machine. Data were submitted to statistical analysis.

**Results:**

After distilled water immersion, nanofilled composite showed the highest value of both flexural strength and elastic modulus, but its flexural values decreased after acidic drink immersion. No significant differences were reported between distilled water and acidic drink immersion for all other materials tested both for flexural and for elastic modulus values.

**Conclusions:**

Even if nanofilled composite showed highest results, acidic drink immersion significantly reduced flexural values.

## 1. Introduction

Dental caries is an infective process that causes the fade of the tooth's hydroxyapatite. This process is mainly due to acid degradation of tooth structures. In order to stop the progression of this disease, the infected tissue has to be removed and it has to be replaced with a filling material [1]. In the past, amalgam was used to replace the infected material, but nowadays dental composites are used to fill the cavity. Unlike amalgam, composites are more difficult to place and they are corroded more easily by the acids of the oral cavity [2–4].

Composites are also used to treat other noncarious lesions of dental tissues, such as in case of dental erosion. This process is an irreversible loss of dental hard tissue by a chemical process without the involvement of microorganisms and this is due to either extrinsic (e.g., high consumption of acid beverages and other acid substances) or intrinsic (e.g., recurrent vomiting due to anorexia and bulimia) sources [5, 6]. In fact, many studies demonstrated that the acids contained in beverages [for example, coca cola] cause the erosion of the enamel, both in vitro and in vivo [7–9]. These acid substances can damage the tissues of the teeth, but also the materials used to restore the teeth [10]. In fact, it is demonstrated that the persistence of the composites into an acidic environment can cause a loss of mechanical properties of composites, glass-ionomer cements, and polyacid modified composites [11, 12]. The creation of microinfiltration between the enamel and the restauration has been reported [13]. Moreover, acidic environment makes the surface of the composites rougher [14]. Many studies evaluated mechanical properties of composites [15, 16]. However, there are no studies about the change of flexural strength and elastic modulus of composite resins after exposure to acidic drinks.

The purpose of the present investigation was to evaluate and compare deflection strengths and elastic modulus of various composites tested after acidic drink immersion.

## 2. Methods

### 2.1. Specimen Preparation

The 9 different composites were divided into groups (Table 1): (1) nanofilled composite (Filtek Supreme XTE, 3M ESPE, St. Paul, Minnesota, USA), (2) microfilled hybrid composite (G-ænial, GC Corporation, Tokyo, Japan), (3) nanohybrid Ormocer based composite (Admira Fusion, Voco, Cuxhaven, Germany), (4) microfilled composite (Gradia Direct, GC Corporation, Tokyo, Japan), (5) microfilled hybrid composite (Essentia, GC Corporation, Tokyo, Japan), (6) nanoceramic composite (Ceram.X Universal, Dentsply De Trey, Konstanz, Germany), (7) supranano spherical hybrid composite (Estelite Asteria, Tokuyama Dental corporation, Taitou-kuTokyo, Japan), (8) microfilled hybrid flowable composite (Gradia Direct Flo, GC Corporation, Tokyo, Japan), and (9) bulk composite (SureFil SDR flow, Dentsply De Trey, Konstanz, Germany).

**Figure 1:**
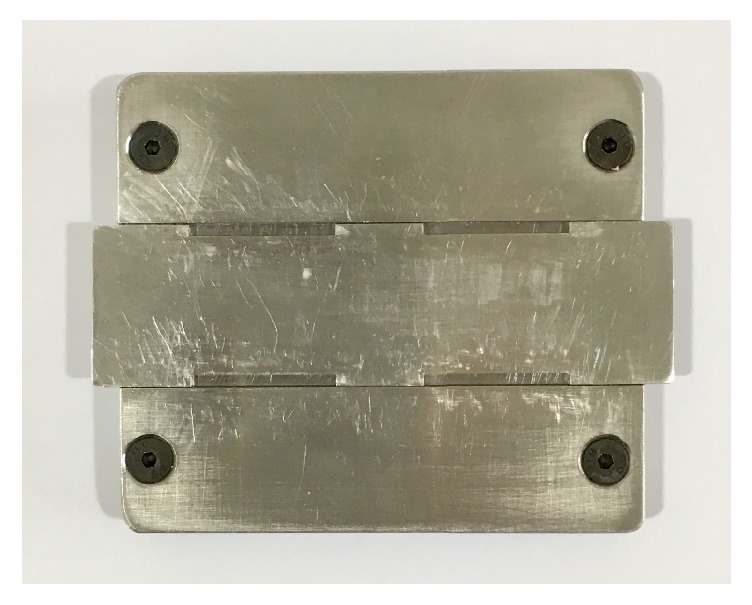
Ad hoc stainless-steel device for specimen preparation.

Thirty rectangular prism-shaped specimens (2mm × 2 mm × 25 mm) of each composite were prepared [17] with ad hoc stainless-steel device (Figure 1). The surface-to-volume ratio was 2,08 mm^−1^. Once the composite was packed into the device, a mylar strip was used to make a flat surface of the specimen. Then all the specimens were light-cured for 3 minutes [18] into a light polymerization oven (Spectramat, Ivoclar Vivadent AG, Schaan, Liechtenstein light intensity: 1200 mW/cm^2^, Wavelength: 430-480 nm, Lamp socket: R7s, Lamp Diameter: 13.5 mm, Lamp length: 160 mm). After polymerization, the specimens were stored in distilled water at 37°C and 100% humidity before performing the flexural strength test.

### 2.2. Immersion in Acidic Drink

Each group of composites (total size for each group: 30 specimens) was divided into three subgroups (A, B, and C) of ten specimens each: the first subgroup (A) was used as control and were tested immediately after storage in distilled water for 24 hours; the specimens of the second subgroup (B) were immersed in 50 ml of acidic drink (Coca Cola/Coca Cola Company, Atlanta, Georgia, United States) for 1 week; the specimens of the last subgroup (C) were immersed in 50 ml of acidic drink (Coca Cola/Coca Cola Company, Atlanta, Georgia, United States) for 1 month. All specimens were immersed at 37°C and 100% humidity environment. A single specimen has been immersed for each of the 50 ml aliquots. The acidic drink was changed once a week for all the time [19]. The pH of acidic storage solution has been measured before specimen immersion (pH=2.4). For subgroup B another pH measurement has been performed before specimen testing (pH=2.4). On the other hand, for subgroup C pH values were recorded each week after solution change (pH=2.4) and before testing (pH=2.4).

### 2.3. Mechanical Test

**Figure 2:**
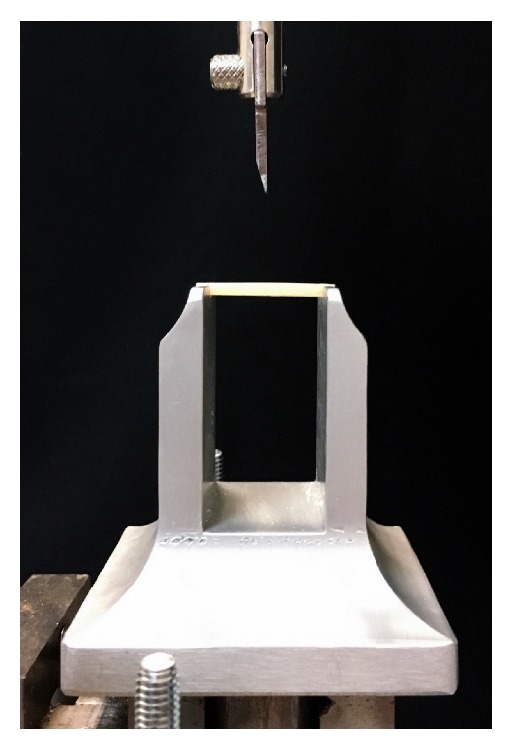
Testing apparatus, before test.

Each sample was placed in an appropriate aluminum framework (Figure 2). The span length between supports was 21 mm and the crosshead speed was set at 1 mm per minute [20]. The compressive load was applied with a universal testing machine (Model 3343, Instron Corporation, Canton, MA, USA) to the middle of the test specimens [21].

**Figure 3:**
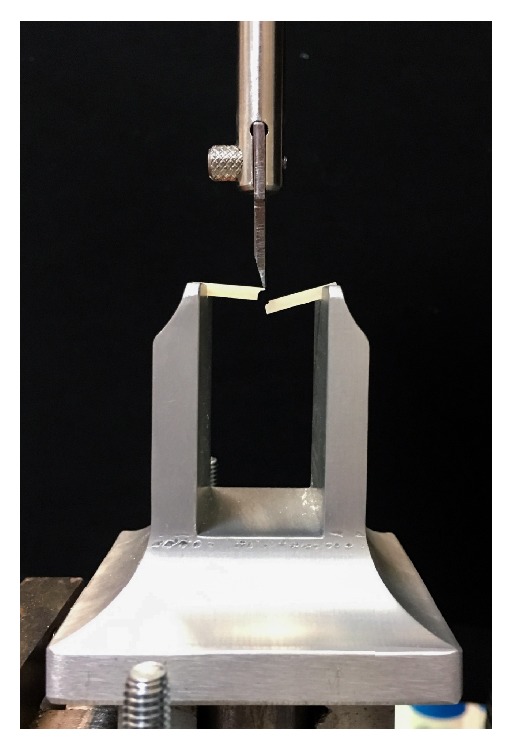
Testing apparatus, after test.

After specimen failure (Figure 3) the flexural strength values were recorded with computer software (Bluehill, Instron Corporation, Canton, Ma, USA).

After collecting the data, the flexural strength (*σ*) and the elastic modulus (E) have been calculated [22].

Statistical analysis was performed with computer software (R version 3.1.3, R Development Core Team, R Foundation for Statistical Computing, Wien, Austria). Descriptive statistics were calculated (mean and standard deviation values). Normality of the distributions was assessed with Kolmogorov and Smirnov test. Nonparametric analysis of variance (Kruskal-Wallis) was applied to determine whether there were significant differences among the various groups. Mann–Whitney post hoc test was applied. Significance for all statistical tests was predetermined at P<0.05.

## 3. Results

### 3.1. Flexural Strength

**Figure 4:**
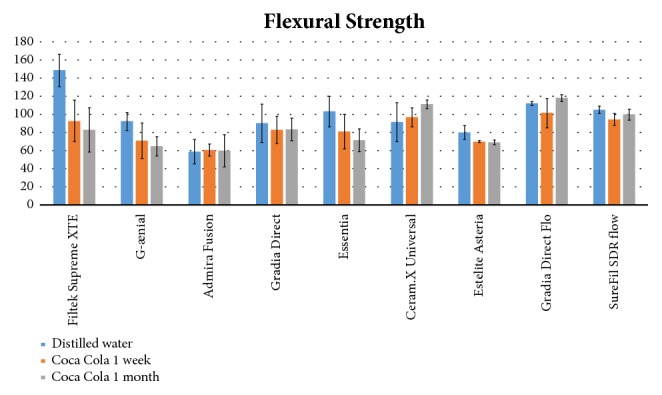
Flexural strength (MPa) for each material tested after 24 hours' distilled water storage (Subgroup A, blue columns), one-week acidic drink storage (Subgroup B, orange columns), and 30 days' acidic drink storage (Subgroup C, grey columns).

Mean and standard deviations of the various groups are illustrated in Table 2 and Figure 4. Kruskal-Wallis showed significant differences among groups (P<0.0001). Post hoc test showed that, after 24 hours' immersion in distilled water (Subgroup A), Filtek Supreme XTE (Group 1) has revealed the highest value of flexural strength if compared with all the composites tested (P<0.05). Admira Fusion (Group 3) recorded the lowest flexural strength values, but the difference was significant only with Essentia (Group 5), Gradia Direct Flo (Group 8), and SureFil SDR flow (Group 9) (P<0.05). The results of the groups that shoved intermediate flexural strength values were as follows: G-ænial (Group 2), Gradia Direct (Group 4), Essentia (Group 5), Ceram.X Universal (Group 6), Estelite Asteria (Group 7), Gradia Direct Flo (Group 8), and SureFil SDR flow (Group 9) showed similar values of flexural strength and no significant difference has been reported among them (P>0.05).

When evaluating the samples of Subgroups B (after 1-week Coca Cola immersion) and C (after 1-month Coca Cola immersion), Filtek Supreme XTE showed a significant decrease in flexural strength if compared with distilled water immersion (Subgroup A) (P<0.05). All the other composites tested showed no significant differences in flexural strength values among the three different storage conditions (P>0.05), except for Filtek Supreme XTE (Group 1) that showed significantly higher values after storage in distilled water than after 30 days of acidic drink storage (P<0.05).

### 3.2. Elastic Modulus

**Figure 5:**
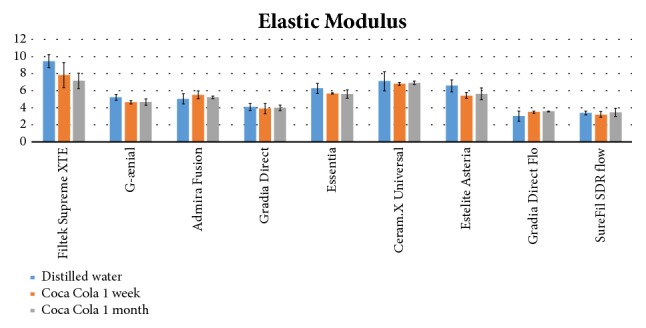
Elastic Modulus (MPa) for each material tested after 24 hours' distilled water storage (Subgroup A, blue columns), one-week acidic drink storage (Subgroup B, orange columns), and 30 days' acidic drink storage (Subgroup C, grey columns).

Mean and standard deviations of the various groups are illustrated in Table 3 and Figure 5. Kruskal-Wallis showed significant differences among groups (P<0.0001). Post hoc test showed that, after 24 hours' immersion in distilled water (Subgroup A), Filtek Supreme XTE (Group 1) showed the highest value of elastic modulus in comparison of all the other composites (P<0.05). Ceram.X Universal (Group 6) showed no significant differences in elastic modulus values if compared with Essentia (Group 5) and Estelite Asteria (Group 7). The lowest values were recorded with Gradia Direct (Group 4), Gradia Direct Flo (Group 8), and SureFil SDR flow (Group 9), which showed no significant difference among them (P>0.05). Gradia Direct (Group 4) showed no significant differences in elastic modulus also if compared with G-Aenial (Group 2) and Admira Fusion (Group 3) (P>0.05).

When evaluating the samples of Subgroups B (after 1-week Coca Cola immersion) and C (after 1-month Coca Cola immersion) similar results were reported, except for Filtek Supreme XTE (Group 1) that showed no significant differences (P>0.05) with Ceram.X Universal (Group 6).

Each of the composites tested showed no significant differences in elastic modulus values among the three different storage conditions (P>0.05), except for Filtek Supreme XTE (Group 1) that showed significantly higher values after storage in distilled water than after both one week and 30 days of acidic drink storage (P<0.05).

## 4. Discussion

Durability of restorations in the oral cavity is highly affected by the resistance to dissolution or disintegration caused by foods, drinks, and the acidity produced by bacteria [23]. Postprandial pH usually becomes more acid as it falls to values below 4. Moreover, many beverages, like Coca Cola, have a value of pH lower than 4. Many scientific studies have shown that enamel dissolution occurs below pH 4 and occurs for the dental composites [5]. Fillers of composite resins have been reported to fall out from resin materials and the matrix component decomposes when exposed to low pH environments [10]. Furthermore, resin-based restorations undergo greater micromorphological damage when remaining in an acidic environment for a long time [14]. In the present study the effect of low pH environment on different dental composites has been evaluated after both one week and 30 days of storage and compared with a control group stored in distilled water for 24 hours. Immersion in distilled water has not been performed after 30 days. In fact, the immersion in water for short term storage periods (within one month) has been demonstrated to have minimal or negligible effect on flexural properties and elastic modulus of composite materials [15]. Moreover, the decrease over time of flexural strength of composite resins stored in water seems to be related more to cyclical fatigue than to storage solution [16]. Our report simulated the acidic environment of the oral cavity by an uninterrupted immersion of the specimens in Coca Cola. However, a limitation of this in vitro model is that other factors have not been considered, such as salivary buffering capacity and acquired pellicle [10]. However, the simulated immersion in acidic drinks has been demonstrated to be a valuable in vitro simulation condition to test composite dental materials [23–25].

In the present report, the flexural strength and elastic modulus of different composite materials have been tested. Previous studies evaluated flexural strength and elastic modulus of various dental materials. Many reports have been presented about fiber-reinforced composites used as endodontic posts [26], nets [27], and long fibers [21]. These materials have been proposed for endodontic [28], prosthodontic [29], and splinting purposes [30]. However, only few reports evaluated flexural strength of nonfiber reinforced dental materials. Only the relationship between flexural strength and the postcuring treatment [17] and the relationship between flexural strength and the different polishing protocols [31] have been tested nowadays. No studies evaluated flexural strength and acidic aging.

The persistence of an acidic environment can cause a loss of mechanical properties of composites, glass-ionomer cements, and polyacid modified composites [11, 12]. In fact, in the present report, the acidic beverage affected only a little the flexural strength of the various composites tested. This is probably due to the different chemistry of the materials tested (composites in the present investigations and compomers or glass ionomer cements in previous reports). In fact, compomers and glass ionomers have the ability to buffer external storage media [11], and this could explain their major susceptibility to acid attack.

In the present report, the most affected composite is the nanofilled composite (Filtek supreme XTE, Group 1), which is also the composite that has reached the highest value of flexural strength under physiologic solution storage. Furthermore, value of flexural strength of nanofilled composite (Group 1) after one month of Coca-Cola reached the average value of all the other composites. Regarding other composites, the effect of the acidic beverage does not affect significantly the flexural strength. Therefore, nanofilled composites present higher flexural strength than all other materials tested without immersion in acidic drink solution. On the other hand, in acidic environment no difference was reported among all materials tested.

It is also possible to notice that Admira Fusion (Group 3) has the lowest value of flexural strength. This is a nanohybrid Ormocer based composite, and it is a different type of composite compared to all of the other composites tested. Ormocer is a composite technology that literally means ORganically MOdified CERamics [32]. These materials are made of inorganic-organic hybrid polymers that form a siloxane network modified by the incorporation of organic groups. This type of composite shows, as a positive aspect, a lower cytotoxic effect than conventional dimethacrylate-based composites [33]. On the other hand, the first generation of ormocers have showed poorer long-term clinical behavior than conventional composites [34]. This is in agreement with the findings of the present report, as the nanohybrid Ormocer based composite (Admira Fusion, Group 3) presented the lowest value of flexural strength compared to other composites tested.

Flexural strength is a clinically relevant property for restorative materials, as it simulates composite use in high-stress bearing areas [35, 36]. Moreover, there is an ISO 4049/2009, which put the limit of 80 MPa for polymer-based restorative materials claimed by the manufacturer as suitable for restorations involving occlusal surfaces [37]. In the present report, under physiologic solution storage, the only material that is not above this ideal value is the nanohybrid Ormocer composite (Group 3). On the other hand, after 1-week acidic drink storage, also microfilled hybrid composite (G-ænial, Group 2) and supranano spherical hybrid composite (Estelite Asteria, Group 7) presented flexural strength values under this limit. After 1 month also microfilled hybrid composite (Essentia, Group 5) decreased its strength performances under 80 MPa. Other materials, such as nanofilled composite (Filtek Supreme XTE, Group 1), microfilled composite (Gradia Direct, Group 4), nanoceramic composite (Ceram.X Universal, Group 6), microfilled hybrid composite (Gradia Direct Flo, Group 8), and bulk fill flowable (SureFil SDR Flow, Group 9), lowered their flexural strength values in acidic environment but remained above the ideal value. This result confirms that acidic environment affects mechanical properties of many restorative materials, including also flexural strength. Indeed, flexural strength is the property that allows composites to resist chewing loads, so this property strongly affects the life of a restauration in the oral cavity [38].

Furthermore, the data collected in the present investigation suggest that the percentage of the filler in the composites presumably does not affect flexural strength. This is in agreement with previous reports evaluating physical properties and depth of cure of composites [35, 39]. Flowable composites, such as flowable microfilled hybrid composite (Gradia Direct Flo, Group 8) and flowable bulk fill (SureFil SDR flow, Group 9), show a higher value of flexural strength compared to supranano spherical hybrid composite (Estelite Asteria, Group 7) and nanohybrid Ormocer based composite (Admira Fusion, Group 3). The latter present a higher percentage of filler. In fact, some authors claimed that there are other factors that can play a relevant role in modifying the flexural strength value such as stress transfer between filler particles and matrix, as well as adhesion between these components [35, 39].

In the present report, also elastic modulus has been tested. Higher values were reported with nanofilled composite (Filtek Supreme XTE, Group 1) and lowest values were reported with microfilled composite (Gradia Direct, Group 4), flowable microfilled hybrid composite (Gradia Direct Flo, Group 8), and flowable bulk fill (SureFil SDR flow, Group 9). As Filtek Supreme XTE (Group 1) showed the highest value of elastic modulus, this material can be considered the stiffest composite tested. The importance of the elastic modulus is mainly related to the choice of the right composite for a specific clinical situation: in fact, flowable composites are usually used in restorations of V class because their higher elasticity can absorb at best the chewing force in this specific region of the tooth. On the other hand, in occlusal restoration, where the material resistance has to be maximized, stiffer composites are more fit to the scope [38, 40]. Previous authors evaluated elastic modulus of different composite materials showing a significant increase of this variable after oven postcuring [17]. No studies evaluated elastic modulus of composites after acidic immersion. In our study the immersion in acidic drink did not affect elastic modulus of all materials tested, so no significant correlation between acidic environment and elastic modulus of composites was found.

Recent research showed a significant increase in elastic modulus of various composite materials, with the increase of filler content [41]. In fact, also in the present report some composites with higher filler percentages showed higher elastic modulus values, as flowable composites tested (groups 8 and 9) showed significantly lower elastic modulus values than higher filler composites (Groups 1 to 7). However, even if the findings of the present report are promising, further in vivo studies are needed in order to confirm the results.

## 5. Conclusions

The results of the present report, showed the following:

(i) The various composite materials showed different flexural strengths and elastic moduli. Higher values were reported with nanofilled composite (Filtek Supreme XTE).

(ii) Immersion in acidic drink lowered flexural strength and elastic modulus of nanofilled composite (Filtek Supreme XTE). Other materials were not affected by acidic drink immersion.

## Figures and Tables

**Table 1:** Materials tested in the present study.

**Material**	**Type**	**Composition**	**Filler Content **%** (w/w)**	**Manufacturer**	**Lot #**
Filtek Supreme XTE	Nanofilled composite	Matrix: Bis-phenol A diglycidylmethacrylate (Bis-GMA), triehtylene glycol dimethacrylate (TEGDMA), urethane dimethacrylate (UDMA), bis-phenol A polyethylene glycol diether dimethacrylate Filler: silica nanofillers (5-75 nm), zirconia/silica nanoclusters (0.6-1.4 *μ*m)	78.5 (w/w)	3M ESPE, St Paul, MN, USA	N748173

G-aenial	Microfilled hybrid composite	Matrix: urethane dimethacrylate (UDMA), dimethacrylate co-monomers. Filler: silica, strontium, lanthanoid fluoride (16-17 *μ*m), silica (>100 nm) fumed silica (<100 nm)	76 w/w	GC Corporation, Tokyo, Japan	151029A

Admira Fusion	Nanohybrid Ormocer based composite	Matrix: resine Ormocer Filler: silicon oxide nano filler, glass ceramics filler (1 *μ*m)	84 (w/w)	Voco, Cuxhaven, Germany	1601121

Gradia Direct	Microfilled composite	Matrix: urethanedimethacrylate (UDMA), dymethacrylate camphorquinone Filler: fluoro-alumino-silicate glass silica powder	73 (w/w)	GC Corporation, Tokyo, Japan	150527A

Essentia	Microfilled hybrid composite	Matrix: urethane dimethacrylate (UDMA), Bis-MEPP, Bis-EMA, Bis-GMA, TEGDMA Filler: prepolymerised fillers, barium glass, fumed silica	81 w/w	GC Corporation, Tokyo, Japan	151109C

Ceram.X Universal	Nanoceramic composite	Matrix: methacrylate modified polysiloxane, dimethacrylate resin, fluorescent pigment, UV stabilizer, stabilizer, camphorquinone, ethyl-4 (dimethylamino) benzoate, iron oxide pigments, aluminium sulfo silicate pigments. Filler: Barium-aluminium borosilicate glass (1.1-1.5 *μ*m), Methacrylate functionalized silicon dioxide nanofiller (10 nm)	76 (w/w)	Dentsply De Trey, Konstanz, Germany	1507000661

Estelite Asteria	Supra-nano spherical hybrid composite	Matrix: Bis-phenol A diglycidylmethacrylate (Bis-GMA), Bisphenol A polyethoxy methacrylate (Bis-MPEPP), triehtylene glycol dimethacrylate (TEGDMA), urethane dimethacrylate (UDMA) Filler: Supra-nano Spherical filler (200nm spherical SiO2-ZrO2), Composite Filler (include 200nm spherical SiO2-ZrO2).	82 (w/w)	Tokuyama Dental corporation, Taitou-kuTokyo, Japan	6,6E+17

Gradia Direct Flo	Flowable Microfilled hybrid composite	Matrix: Modified UDMA, EBPADMA, TEGDMA Filler: Ba–Al–F–B–Si–glass, St–Al–F–Si–glass 68 wt%, 44 vol%		GC Corporation, Tokyo, Japan	160602A

SureFil SDR flow	Bulk Fill Flowable	Matrix: Modified UDMA, EBPADMA, TEGDMA Filler: Barium-alumino-fluoro-borosilicate glass, strontium alumino-fluoro-silicate glass 47,3 vol%		Dentsply De Trey, Konstanz, Germany	1703001234

**Table 2:** Mean values (and standard deviations) of flexural strength (MPa). Values have been collected after storage in physiologic solution (Subgroup A, t0), after one week (Subgroup B, t1), and after 30 days (Subgroup C, t2) of immersions in acidic drink.

	**Group 1 (Filtek Supreme XTE)**	**Group 2 (G-ænial)**	**Group 3 (Admira Fusion)**	**Group 4 (Gradia Direct)**	**Group 5 (Essentia)**	**Group 6 (Ceram.X Universal)**	**Group 7 (Estelite Asteria)**	**Group 8 (Gradia Direct Flo)**	**Group 9 (SureFil SDR flow)**
**Subgroup A (t0)**	148,58 (17,77)	92,31 (9,85)	58,88 (13,54)	90,21 (21,27)	103,22 (16,90)	91,67 (21,69)	80,29 (7,58)	112,27 (2,31)	105,16 (4,08)

**Subgroup B (t1)**	93,07 (22,84)	70,94 (19,55)	60,66 (6,81)	82,98 (14,94)	81,14 (19,24)	96,93 (10,56)	70,11 (1,11)	101,61 (16,18)	94,57 (6,64)

**Subgroup C (t2)**	82,98 (24,26)	64,86 (10,90)	60,07 (17,78)	83,55 (12,41)	71,52 (12,50)	111,14 (4,84)	69,23 (2,39)	118,40 (3,77)	99,89 (5,98)

**Table 3:** Mean values (and standard deviations) of elastic modulus (GPa). Values have been collected after storage in physiologic solution (Subgroup A, t0), after one week (Subgroup B, t1) and after one month (Subgroup C, t2) of immersions in acidic drink.

	**Group 1 (Filtek Supreme XTE)**	**Group 2 (G-ænial)**	**Group 3 (Admira Fusion)**	**Group 4 (Gradia Direct)**	**Group 5 (Essentia)**	**Group 6 (Ceram.X Universal)**	**Group 7 (Estelite Asteria)**	**Group 8 (Gradia Direct Flo)**	**Group 9 (SureFil SDR flow)**
**Subgroup A (t0)**	9,45 (0,78)	5,25 (0,35)	5,06 (0,59)	4,11 (0,38)	6,27 (0,57)	7,12 (1,12)	6,57 (0,68)	3,05 (0,60)	3,40 (0,23)
**Subgroup B (t1)**	7,83 (1,47)	4,65 (0,19)	5,52 (0,43)	3,90 (0,59)	5,70 (0,05)	6,81 (0,19)	5,45 (0,33)	3,50 (0,15)	3,22 (0,34)
**Subgroup C (t2)**	7,16 (0,89)	4,68 (0,37)	5,23 (0,15)	4,00 (0,33)	5,60 (0,49)	6,92 (0,20)	5,65 (0,69)	3,60 (0,04)	3,49 (0,47)

## Data Availability

Data are available upon request at andrea.scribante@unipv.it.

## References

[1] Dahl B. L., Carlsson G. E., Ekfeldt A. (1993). Occlusal wear of teeth and restorative materials: a review of classification, etiology, mechanisms of wear, and some aspects of restorative procedures. *Acta Odontologica Scandinavica*.

[2] Bernardo M., Luis H., Martin M. D. (2007). Survival and reasons for failure of amalgam versus composite posterior restorations placed in a randomized clinical trial. *The Journal of the American Dental Association*.

[3] Mehdawi I. M., Pratten J., Spratt D. A., Knowles J. C., Young A. M. (2013). High strength re-mineralizing, antibacterial dental composites with reactive calcium phosphates. *Dental Materials*.

[4] Krifka S., Hiller K.-A., Bolay C. (2012). Function of MAPK and downstream transcription factors in monomer-induced apoptosis. *Biomaterials*.

[5] Kitchens M., Owens B. M. (2007). Effect of carbonated beverages, coffee, sports and high energy drinks, and bottled water on the in vitro erosion characteristics of dental enamel. *Journal of Clinical Pediatric Dentistry*.

[6] Pedrini D., Candido M. S. M., Rodrigues Jr. A. L. (2003). Analysis of surface roughness of glass-ionomer cements and compomer. *Journal of Oral Rehabilitation*.

[7] Larsen M., Nyvad B. (1999). Enamel erosion by some soft drinks and orange juices relative to their pH, buffering effect and contents of calcium phosphate. *Caries Research*.

[8] West N. X., Maxwell A., Hughes J. A., Parker D. M., Newcombe R. G., Addy M. (1998). A method to measure clinical erosion: The effect of orange juice consumption on erosion of enamel. *Journal of Dentistry*.

[9] Meurman J. H., Frank R. M. (1991). Scanning electron microscopic study of the effect of salivary pellicle on enamel erosion. *Caries Research*.

[10] Rajavardhan K., Sankar A., Kumar M., Kumar K., Pranitha K., Kishore K. (2014). Erosive potential of cola and orange fruit juice on tooth colored restorative materials. *Annals of Medical and Health Sciences Research*.

[11] Aliping-Mckenzie M., Linden R. W. A., Nicholson J. W. (2004). The effect of Coca-Cola and fruit juices on the surface hardness of glass-ionomers and ‘compomers’. *Journal of Oral Rehabilitation*.

[12] De Paula A. B., De Fúcio S. B. P., Alonso R. C. B., Ambrosano G. M. B., Puppin-Rontani R. M. (2014). Influence of chemical degradation on the surface properties of nano restorative materials. *Operative Dentistry*.

[13] Katge F., Shitoot A., Pammi T., Mithiborwala S. (2016). Evaluation of microleakage of nanoionomer and nanocomposite restorations, immersed in fruit drink, fresh fruit juice and soft drink -An in vitro study. *Journal of Clinical Pediatric Dentistry*.

[14] Abu-Bakr N. H., Han L., Okamoto A., Iwaku M. (2000). Changes in the mechanical properties and surface texture of compomer immersed in various media. *Journal of Prosthetic Dentistry*.

[15] Drummond J. L., Lin L., Al-Turki L. A., Hurley R. K. (2009). Fatigue behaviour of dental composite materials. *Journal of Dentistry*.

[16] Hurley R. K., Drummond J. L., Viana G. C., Galang M. T. (2012). The effects of environment and cyclic fatigue on the mechanical properties of an indirect composite. *Journal of Dentistry*.

[17] Almeida-Chetti V. A., Macchi R. L., Iglesias M. E. (2014). Effect of post-curing treatment on mechanical properties of composite resins. *Acta Odontológica Latinoamericana*.

[18] Cacciafesta V., Sfondrini M. F., Lena A., Scribante A., Vallittu P. K., Lassila L. V. (2007). Flexural strengths of fiber-reinforced composites polymerized with conventional light-curing and additional postcuring. *American Journal of Orthodontics and Dentofacial Orthopedics*.

[19] Briso A. L. F., Caruzo L. P., Guedes A. P. A., Catelan A., Dos Santos P. H. (2011). In vitro evaluation of surface roughness and microhardness of restorative materials submitted to erosive challenges. *Operative Dentistry*.

[20] Scribante A., Massironi S., Pieraccini G. (2015). Effects of nanofillers on mechanical properties of fiber-reinforced composites polymerized with light-curing and additional postcuring. *Journal of Applied Biomaterials and Functional Materials*.

[21] Sfondrini M. F., Massironi S., Pieraccini G. (2014). Flexural strengths of conventional and nanofilled fiber-reinforced composites: a three-point bending test. *Dental Traumatology*.

[22] Rodrigues Jr. S. A., Zanchi C. H., de Carvalho R. V., Demarco F. F. (2007). Flexural strength and modulus of elasticity of different types of resin-based composites. *Brazilian Oral Research*.

[23] Han L., Okamoto A., Fukushima M., Okiji T. (2008). Evaluation of flowable resin composite surfaces eroded by acidic and alcoholic drinks. *Dental Materials*.

[24] Lepri C. P., Palma-Dibb R. G. (2012). Surface roughness and color change of a composite: Influence of beverages and brushing. *Dental Materials*.

[25] Poggio C., Dagna A., Chiesa M., Colombo M., Scribante A. (2012). Surface roughness of flowable resin composites eroded by acidic and alcoholic drinks. *Journal of Conservative Dentistry*.

[26] Irmak Ö., Yaman B. C., Lee D. Y., Orhan E. O., Mante F. K., Ozer F. (2018). Flexural strength of fiber reinforced posts after mechanical aging by simulated chewing forces. *Journal of the Mechanical Behavior of Biomedical Materials*.

[27] Sfondrini M. F., Cacciafesta V., Scribante A. (2011). Shear bond strength of fibre-reinforced composite nets using two different adhesive systems. *European Journal of Orthodontics*.

[28] Özcan E., Çetin A. R., Çapar I. D., Tunçdemir A. R., Aydinbelge H. A. (2013). Influence of eugenol on the push-out bond strengths of fiber posts cemented with different types of resin luting agents. *Odontology*.

[29] Vallittu P. K., Shinya A., Baraba A. (2017). Fiber-reinforced composites in fixed prosthodontics—Quo vadis?. *Dental Materials*.

[30] Scribante A., Sfondrini M. F., Broggini S., D'Allocco M., Gandini P. (2011). Efficacy of esthetic retainers: clinical comparison between multistranded wires and direct-bond glass fiber-reinforced composite splints. *International Journal of Dentistry*.

[31] Ramirez-Molina R., Kaplan A. E. (2015). Influence of polishing protocol on flexural properties of several dental composite resins. *Acta Odontológica Latinoamericana*.

[32] Hakim F., Vallée J. (2018). Use of a novel ORMOCER as a universal direct restorative material. *Compendium of Continuing Education in Dentistry*.

[33] Susila A. V., Balasubramanian V. (2016). Correlation of elution and sensitivity of cell lines to dental composites. *Dental Materials*.

[34] Monsarrat P., Garnier S., Vergnes J.-N., Nasr K., Grosgogeat B., Joniot S. (2017). Survival of directly placed ormocer-based restorative materials: A systematic review and meta-analysis of clinical trials. *Dental Materials*.

[35] Garoushi S., Säilynoja E., Vallittu P. K., Lassila L. (2013). Physical properties and depth of cure of a new short fiber reinforced composite. *Dental Materials*.

[36] Finan L., Palin W. M., Moskwa N., McGinley E. L., Fleming G. J. P. (2013). The influence of irradiation potential on the degree of conversion and mechanical properties of two bulk-fill flowable RBC base materials. *Dental Materials*.

[37] ISO4049:2009 (2009). *Dentistry-resin based filling materials*.

[38] Sooraparaju S. G., Kanumuru P. K., Nujella S. K., Konda K. R., Reddy K. B. K., Penigalapati S. (2014). A comparative evaluation of microleakage in class v composite restorations. *International Journal of Dentistry*.

[39] Goracci C., Cadenaro M., Fontanive L. (2014). Polymerization efficiency and flexural strength of low-stress restorative composites. *Dental Materials*.

[40] Yazici A. R., Baseren M., Dayangaç B. (2003). The effect of flowable resin composite on microleakage in class V cavities. *Operative Dentistry*.

[41] Yamamoto T., Hanabusa M., Kimura S., Momoi Y., Hayakawa T. (2018). Changes in polymerization stress and elastic modulus of bulk-fill resin composites for 24 hours after irradiation. *Dental Materials*.

